# A Balanced Look at the Implications of Genomic (and Other “Omics”) Testing for Disease Diagnosis and Clinical Care

**DOI:** 10.3390/genes5030748

**Published:** 2014-09-01

**Authors:** Scott D. Boyd, Stephen J. Galli, Iris Schrijver, James L. Zehnder, Euan A. Ashley, Jason D. Merker

**Affiliations:** 1 Department of Pathology, Stanford University, Stanford, CA 94305, USA; E-Mails: sgalli@stanford.edu (S.J.G.); ischrijver@stanfordmed.org (I.S.); zehnder@stanford.edu (J.L.Z.); jdmerker@stanford.edu (J.D.M.); 2 Department of Microbiology and Immunology, Stanford University, Stanford, CA 94305, USA; 3 Department of Pediatrics, Stanford University, Stanford, CA 94305, USA; 4 Department of Medicine, Stanford University, Stanford, CA 94305, USA; E-Mail: euan@stanford.edu

**Keywords:** personalized medicine, clinical genomics, regulation, quality assurance

## Abstract

The tremendous increase in DNA sequencing capacity arising from the commercialization of “next generation” instruments has opened the door to innumerable routes of investigation in basic and translational medical science. It enables very large data sets to be gathered, whose interpretation and conversion into useful knowledge is only beginning. A challenge for modern healthcare systems and academic medical centers is to apply these new methods for the diagnosis of disease and the management of patient care without unnecessary delay, but also with appropriate evaluation of the quality of data and interpretation, as well as the clinical value of the insights gained. Most critically, the standards applied for evaluating these new laboratory data and ensuring that the results and their significance are clearly communicated to patients and their caregivers should be at least as rigorous as those applied to other kinds of medical tests. Here, we present an overview of conceptual and practical issues to be considered in planning for the integration of genomic methods or, in principle, any other type of “omics” testing into clinical care.

## 1. Introduction

Improvements in DNA sequencing technology in the past decade represent one of the most significant technological achievements in recent history, with far-reaching implications for medicine and society. Most human diseases have at least some genetic factors that contribute to their incidence, or course, either related to the germline genome inherited from an individual’s parents or the somatic genetic changes that can lead to the development of malignancies. The ability to read the DNA from an individual’s cells with next generation sequencing (NGS) should therefore offer insights relevant for medical care. However, there is a significant gap between our current ability to acquire sequence data and the ultimate goal of extracting all of the useful medical genetic knowledge from the sequences. In particular, societal expectations and ethical considerations require that any correlations between DNA sequences and predictions of disease risk, prognosis or optimal treatment choice should be held to higher standards of evidence than those that are typically applied in the peer review process for publication of a research article. In this overview, we initially highlight areas of recent progress and promise in clinical genomic testing (including whole genome sequence analysis, as well as analysis of selected fractions of the genome, such as the protein coding exome, or large panels of genes of clinical interest) and discuss these new approaches in the context of medical laboratory testing and the current regulatory framework governing such tests in the United States. The potential benefits of clinical genomic testing are tremendous, but devising appropriate systems for quality assurance, data sharing and validation, incorporation into clinical trials and cost-benefit analysis of this new diagnostic area will be an ongoing effort in the coming years.

## 2. The Promise of Genomic Methods

In the past decade, the quantity of DNA sequencing that can be performed per dollar spent has increased by several orders of magnitude, as a result of technological innovations enabling highly parallelized simultaneous sequencing of millions of spatially-separated template molecules, with optical or electronic readout of the sequencing reaction as it occurs [1,2]. With the latest generation of sequencing instruments, the cost for a whole human genome sequence with 30× coverage may approach $1,000 [3]. Although such estimates notably exclude the costs of interpreting the data, it is clear that genome sequencing is now within the range of costs for many other diagnostic methods, such as radiologic imaging studies or full evaluation of tissue biopsies by anatomic pathologists. As a result of painstaking earlier studies of the genetics of inherited diseases and the genetic changes that are found in cancer cells, there are already many known gene mutations whose significance in relation to particular diseases is described. For many of the most well-established variants, there are single-gene diagnostic sequencing tests already available, either from private companies or specialized, typically academic, diagnostic laboratories. The CDC estimates that genetic tests for use in the clinical setting have been developed for approximately 2,000 diseases [4]. The critical difference between current genome sequencing capabilities and these earlier test methods is that a significantly greater amount of data (whether genome, exome or gene panels) now can be gathered as readily and easily as the sequence from a single gene. Many medical centers and companies are hurrying to stake claims as the preferred destination for testing and interpretation of genomic sequence data for clinical purposes, as this methodology has begun to be adopted and standardized. 

Already, there are multiple published medical success stories using these methods. Sequencing of genomes or exomes (which includes the protein coding portions of the genome) for the diagnosis of patients with heritable syndromic disorders has resulted in a number of exciting case reports and studies of patients in whom likely causative mutations have been discovered, and in some cases, such findings have guided successful clinical treatment decisions [5,6,7]. Systematic efforts in the NIH Undiagnosed Diseases Program to apply genomic methods to arrive at diagnoses for patients with unusual or mysterious clinical presentations, particularly for cases where family history suggests a possible genetic cause, have also yielded new causative mutations and discoveries in human biology [8]. Likewise, clinical laboratories have used exome sequencing since 2011 to evaluate patients with suspected genetic disorders and have identified a molecular diagnostic yield of approximately 25% [9].

Sequencing of cancer genomes has revealed many new recurrent mutations that may contribute to the development of particular cancers, and has revealed new candidates for targeted therapies [10]. These new molecular insights into cancer are already beginning to influence the ways that cancers are classified and treated [11]. Similarly, sequencing of fetal DNA from the plasma of a pregnant woman to screen for trisomies 18 and 21 demonstrates improved performance relative to prior aneuploidy screening methods [12]. Studies of viruses, bacteria and other microbes via sequencing of their small genomes is revolutionizing epidemiological tracking of infectious diseases and the rapid detection of new and emerging pathogens [13]. The most challenging area of all, predicting the risks of diseases in healthy human beings, has also show promise in particular disease categories, such as prediction of cancer risk in women having germline mutations in the *BRCA1* or *BRCA2* genes. More broadly, it is likely that only a fraction of potential disease-associated variants have been identified at present, and the cooperative or competitive relationships between the effects of different sequence variants are only beginning to be described [14]. Many common diseases (e.g., diabetes, schizophrenia and autism) do not appear to be the result of simple sequence alterations that could be easily diagnosed by DNA sequencing. In these diseases, there are many genes associated with increased risk, but the conditions may be caused by combinations of these genes acting together in association with environmental factors. In addition, while it is now possible to identify numerous variants in cancer genomes, the biological significance of many variants is unknown, and their annotation is not standardized. The number of clinically actionable variants, at present, is small.

However, even given the above limitations, these advances herald the increasing importance that genome sequencing and related methods are likely to play in the diagnosis and management of diseases across all medical specialties. We are currently in a transition period in which methods initially applied in research settings and limited small clinical studies need to be adapted for application to large numbers of patients. With that transition comes a requirement for increased standardization, reliability and monitoring of experimental steps, as well as agreed-upon standards for data analysis, storage, clinical interpretation and communication with patients and/or their guardians.

## 3. Research Experiments, Clinical Testing and Genomic Testing

### 3.1. Research Assays and Methods

The experimental methods used in medical testing typically originate in research laboratories in universities, government or private research institutes or corporations. However, there is a substantial difference between the assay performance characteristics needed for use in the published scientific literature, compared to those used for clinical diagnosis and guiding the treatment of patients. Authors using a new experimental method as part of a published peer-reviewed research study must convince the scientific reviewers selected by the journal that the assay is a valid method of measurement and has been appropriately applied to the research topic in question, but often, reviewers are not experts in all aspects of the experimental methodology and data analysis approaches used. The expectation in a research setting is that efforts by other researchers to replicate the results in question and to use the methods described will eventually test the reported results and reveal any limitations or errors. This approach has been the basis for essentially all scientific advances, despite the fact that very few published papers are entirely free from errors or only partially correct conclusions, and some are entirely false [15].

Recent studies have highlighted common problems with experimental design and statistical analysis that contribute disproportionately to preventable errors in the scientific literature, especially in attempting to identify genetic contributions to diseases [16,17]. These include choices in experimental design that can introduce biases favoring the discovery of apparently significant effects, even in randomized trials, where patient selection, problems with randomization, lack of blinded data analysis and changes in the plan for data analysis once a trial is underway can all have an impact. In case-control or retrospective studies, the potential for mistaken conclusions is even greater. Studies using large data sets are particularly vulnerable to errors from over-fitting a model to the data or insufficiently accounting for multiple hypothesis testing, especially if independent validation data sets are not used to test the robustness of initial conclusions. Other well-known factors that can distort the scientific literature are publication biases in favor of positive results and the competitive social and economic factors that disproportionately reward scientists publishing papers reporting apparently highly novel findings in prominent journals, while imposing minimal penalties for prior publications later found to be partly or completely in error.

Several suggested improvements to the research methodology have been proposed, including advance registration of a wider range of clinical studies, better documentation of experimental protocols, results and data analysis approaches, more consistent involvement of statisticians and other experts in study design and analysis, full transparency and availability of experimental data and computer scripts used in data analysis and greater attention paid to research reproducibility in the professional evaluation of scientists [16]. In practice, an excessively regulated research environment would probably serve to stifle and limit some of the creative and perhaps poorly planned, but ultimately serendipitous efforts in science that can lead to unexpected insights, so a compromise between higher standards for clinical trial research and continued freedom of inquiry (subject to ethical and safety review by institutional review boards) in more exploratory areas of medical science would probably best serve the public and the ultimate goals of funding providers. 

### 3.2. Clinical Tests

Clinical laboratory testing in the United States is regulated and subjected to greater methodological scrutiny than basic or clinical research. All clinical laboratory testing done for purposes of patient care (as opposed to research) must be performed in a CLIA (Clinical Laboratory Improvement Amendments of 1988)-certified laboratory. CLIA established key laboratory quality standards to ensure that test performance consistently meets patient care needs. CLIA certification may be achieved through the Centers for Medicare and Medicaid Services (CMS), which administers CLIA laboratory certification, or through CMS-approved accrediting organizations (e.g., the College of American Pathologists (CAP), or The Joint Commission). CLIA requirements are stratified according to the complexity of testing performed, with genomic testing generally falling into the highest level of complexity. Such high-complexity testing laboratories must meet specified quality standards, including those related to personnel qualifications and responsibilities, proficiency testing, facilities, general laboratory systems and quality management, as well as preanalytic, analytic and postanalytic systems. (CLIA Brochure, ICN #006270, May 2013). Laboratory compliance with CLIA regulations is evaluated by biennial on-site inspections. Depending on the nature of the tests performed, additional requirements may need to be satisfied for such laboratories, including those of the AABB (formerly, the American Association of Blood Banks) (Bethesda, MD, USA), FDA (U.S. Food and Drug Administration, Silver Spring, MD, USA), ASHI (the American Society for Histocompatibility and Immunogenetics, Mt. Laurel, NJ, USA), FAA (Federal Aviation Administration, Washington, DC, USA) and state agencies. High-complexity testing must be done by or subject to the oversight of laboratory professionals with an advanced degree and with appropriate credentials in laboratory medicine. 

New types of laboratory tests, including most genomic tests, are not available in the form of FDA approved/cleared test kits, but rather are typically created within academic or commercial clinical laboratories as laboratory-developed tests (LDTs). Such development follows a strictly prescribed process of test validation prior to clinical implementation, which is when the test becomes orderable [18,19]. Recently, one high-throughput sequencing instrument and reagent kit system has received FDA clearance for use in clinical testing. In a recent position statement, the Association for Molecular Pathology (AMP) has introduced the term, laboratory-developed procedure, which is defined as “a professional service that encompasses and integrates the design, development, validation, verification, and quality systems used in laboratory testing and interpretative reporting in the context of clinical care” and which much more accurately reflects the highly complex nature of molecular laboratory testing, as well as the central contribution of highly trained and qualified laboratory professionals to the patient care process. AMP also concluded that CMS can ensure the effective oversight and validation of most molecular genetic laboratory tests [20].

### 3.3. Genomic Testing

The nature of genomic testing, in which large data sets of DNA sequences can be conveniently gathered, but where only a fraction of the overall data can be interpreted at present, places these data sets at the interface between research and conventional clinical testing. Interpretation of genome sequence data is not unique in requiring sophisticated understanding, both of the methods used to gather the data, and the medical literature and body of prior investigation, to arrive at accurate conclusions. For example, histologic diagnosis of cancers in modern pathology practice depends on years of training in recognizing the visual characteristics of aberrant cell populations in tissue sections and selecting appropriate confirmatory tests. The history of revised and improved tumor classification systems in modern oncology reflects the increased understanding gained over decades by studying tumors with new experimental methods, revisiting prior data and correlating the features of each cancer with its response to treatment. Genomic sequence data are vast and complex in other ways, in that the data are generated as lists of nucleotide identities that require computational tools and comparison to sequences in reference databases for analysis, before the clinical significance of sequence variants can begin to be assessed. 

Despite amazing progress in the past decade, the technologies and analytical approaches for sequencing and interpreting genomes still have significant blind spots, such as the greater difficulty of detecting structural variants including insertions, deletions, inversions, and trinucleotide repeat expansions, compared to single nucleotide variants in sequences [21]. Reference databases of sequence variants also contain many artifactual annotations, such as ‘variants’ that are actually sequences derived from pseudogenes similar to the gene in question. It is likely to take many years to resolve such ambiguities or errors in prior gene sequencing work and to ensure that all sequence variants can be correctly annotated for medical applications. Judging from earlier work in human genetics, the interpretation of each patient’s genome sequence will require a personalized approach that takes into account the population group from which that person is derived. Even for the most extensively studied genes, such as the cystic fibrosis transmembrane conductance regulator (*CFTR*), the databases of mutations and their significance are quite limited, because functional studies have not been performed for most mutations and because mutations in population groups that have not been studied as extensively as European-derived groups are not well characterized [22].

In the near future, we should not necessarily expect that any given patient’s genome sequence, particularly if they are currently healthy, will reveal sequence features requiring any sort of response or clinical guidance beyond those that would already be provided by a physician in a routine checkup, such as advice about healthy diet, exercise, vaccinations and safety topics. A recent study of the potential and current limitations of whole genome sequence interpretation for clinical use in 12 healthy individuals highlighted the relatively small effects associated with most known disease-associated sequence variants, but also revealed that each individual had at least one gene variant from the Clinical Pharmacogenomics Implementation Consortium list of variants that can affect responses to drug therapies [14]. In addition, two of the 12 subjects studied received actionable information that could affect their future health, including one subject who underwent prophylactic surgery. Other studies have underlined the relatively small effect sizes of genetic variants for the most common and serious diseases, such as cardiovascular disease, but rare and highly deleterious variants can also be identified, such as those causing familial hypercholesterolemia, which would warrant immediate medical intervention, such as statin therapy [23,24]. Recently, standards of evidence for concluding that gene variants are associated with diseases have been proposed, taking into account the large amount of data gathered in genome or exome studies and the potential for false discoveries associated with such large numbers of observations of a sample; for example, in exome studies, *p* = 5 × 10^−7^ has been suggested as one threshold for claiming significance [25,26].

## 4. Quality Assurance in the Genome Sequencing Era

Molecular diagnostic laboratories have provided innovative testing since the emergence of diagnostic methods applied to DNA or RNA molecules several decades ago. Clinical molecular laboratories are experienced in validating new methods for single gene testing and data analysis. In one sense, genomic testing is “just another” such innovation. However, it could be argued that the scale of data now obtainable represents a challenge for analysis that is not merely incremental, but is qualitatively different, as human beings cannot manually go through the sequences and interpret them visually in a practical amount of time. Instead, computational methods and bioinformatics tools are required to help carry out the analysis. This represents a significant break with prior traditions of medical training in all specialties, where, with rare exceptions, computer science and bioinformatics were not learned by the generations of physicians who are currently in practice and in positions of authority. Physicians currently in training, particularly in laboratory medicine, now have the opportunity to learn and help to develop these new methods before they enter practice, and currently practicing physicians must at least learn how the results of genomic testing should be applied in their patient care decisions [27,28]. In spite of these challenges, the fundamental features required for reliable clinical laboratory tests based on genome sequencing are the same as those for other kinds of tests and are, in our view, compatible with current regulatory frameworks for ensuring the quality of medical laboratory testing. Some of the most critical initiatives underway in this area are as follows:

*(a) Guidelines for Clinical NGS Implementation*
  Initial laboratory guidelines for clinical diagnostic NGS testing have been established and published by several laboratory professional organizations. These include initiatives by professional organizations, such as the Association for Molecular Pathology (AMP) [29] and the American College of Medical Genetics and Genomics (ACMG) [30,31], as well as those by entities, such as the Clinical Laboratory Standards Institute (CLSI) and the Division of Laboratory Science and Standards at the Centers for Disease Control (CDC) [32]. These efforts are expected eventually to result in consistent recommendations for the clinical validation process of NGS testing, as well as for performance metrics and genomic reference materials for clinical use.
*(b) Checks and Balances: The College of American Pathologists Checklist for NGS Testing*
  The College of American Pathologists (CAP), a CMS-approved accrediting organization, has recently developed a new set of checklist requirements that are specific to NGS, which advances greater standardization in clinical NGS testing. CAP checklists are available to subscribing laboratories and cover key aspects of laboratory function: policies, procedures and pre-analytical, analytical and post-analytical aspects of clinical testing. There is a customized checklist for every section of a clinical laboratory, as well as a general checklist that applies to all sections. During a laboratory inspection, CAP inspectors use these checklists in their evaluation process, to assess whether laboratories follow regulations and practice guidelines and operate at a quality level that is worthy of CAP accreditation and CLIA certification. The NGS section of the molecular checklist contains a set of requirements for both the analytical wet bench processes, as well as for the various bio-informatics steps required for data analysis and annotation. Even though these requirements are not rigidly prescriptive, they highlight key points that must be considered when documenting the reliability and usefulness of clinical NGS testing methods. Many medical centers are within the second year or third of carrying out such testing and therefore have undergone inspection of their genomic or next-generation DNA sequencing assays by CAP or other groups that carry out inspections of CLIA-certified laboratories. Feedback from inspectors and participant laboratories will be very useful for identifying the areas in which checklists need to be revised or made more detailed, explicit or prescriptive, as well as for highlighting the more difficult or uncertain areas in genome sequence data gathering, interpretation and reporting. Some evaluation of the thoroughness of inspections in this new area and the knowledge and qualifications of the inspectors selected for this task will also be warranted as part of the laboratory medicine profession’s due diligence in incorporating these new testing methods into mainstream clinical testing.
*(c) Assay Validation Requirements*
  The assay validation conducted before a test is offered clinically documents that a test is consistently and accurately detecting what it claims to be able to identify. CLIA regulations (Code of Federal Regulations § 493.1253 (b) (2)) stipulate that certain core analytical characteristics must be assessed and documented. These include accuracy, precision, analytical sensitivity, analytical specificity, reportable range, reference intervals (normal values) and any other performance characteristic required for test performance (e.g., carryover, dilutions and calculations). The same parameters should be applied to NGS testing, which ranges in scope from single genes or mutation panels to genome sequencing. Limitations of sequence library generation and interpretation, including poorer quality analysis of repetitive sequence regions, less reliable detection or inability to detect certain categories of variation (e.g., insertions, deletions, and other structural variants), inadequately covered regions and similar problems should be reflected and noted in descriptions of the testing method. During the validation process, every single step of NGS must be evaluated, including sample library preparation, clonal fragment amplification, sequencing and all steps of the analysis.
  A key need for NGS assay development and validation is the availability of well-characterized “gold-standard” reference materials. Fortunately, there are several public efforts and commercial products that are beginning to meet this need. As an example, the National Institute of Standards and Technology (NIST), with the Genome in a Bottle Consortium, has developed well-characterized single genome reference material for SNVs and small insertions and deletions [33]. Continued support of these and related efforts are needed to generate additional reference genomes and other reference materials for additional applications (e.g., somatic variants).
*(d) Interpretation and Reporting of NGS Results*
  The ACMG has previously published recommendations for the interpretation and reporting of sequence variations for heritable disease, and updates to these recommendations that include interpretation and reporting of NGS-derived sequence changes are expected to be released soon [34]. Additional recommendations will likely be required for other applications (e.g., somatic mutation testing in cancer, pharmacogenetic variation). Despite the advances in sequencing technology, many of the key principles of interpretation still apply. SNP databases and disease-related collections of sequence variants are immensely helpful in variant annotation and interpretation, but there are significant issues that prevent them from being reliably used for clinical diagnostic purposes. Many population databases contain individuals that have developed or will develop disease, and many of the disease-specific databases include benign variants. This underscores the importance of centralized efforts to generate clinical-grade variant databases, such as ClinVar [35].
  The final formal interpretation of NGS results, their official posting into the patient’s medical record and their translation into clinical care by the physicians responsible for doing that requires interdisciplinary collaboration, whereby pathologists, geneticists and other laboratory professionals become even more directly involved with others in the healthcare team, in order to ensure accurate diagnostic information for individual patients in the context of their disease phenotype.
*(e) Proficiency Testing for NGS Assays*
  Apart from the creation of NGS-specific checklist items, the CAP is in the process of developing NGS proficiency testing products, which are expected to become available in the near future. No other NGS proficiency testing is available in the U.S. from CMS or a CMS-approved accrediting organization. However, laboratories are required to participate in proficiency testing at least twice per year, and this requirement is currently met by alternative assessment. The purpose of such proficiency testing is to be a central quality assessment tool that is an integral component of laboratory inspections and regulatory requirements. To this end, laboratories commonly perform a blinded proficiency testing exchange with other laboratories.

### Assessing the Utility of Genomic Information in Clinical Patient Care

We anticipate that NGS technologies will continuously improve in their ability to detect sequence changes and will increase their overall accuracy and ease of use. The increasing capabilities and enhancements to these instruments will facilitate the clinical use of genomic data. The information that is returned to the patient, however, reaches further than the technical and interpretational aspects alone and includes the perceived and, therefore, subjective value of the information. For individual patients, therefore, there is the aspect of personal usefulness (value from the patient’s perspective), as well as clinical utility, which constitutes the net health benefits or the balance of benefit* versus* harm. Clinical utility is a complex metric that includes a variety of aspects, such as the patient population tested, the clinical manifestations of the finding and the rationale for testing. Currently, some clinical questions that are explored with NGS can be addressed with considerable confidence, whereas others reach beyond established knowledge. This includes an assessment of the pathogenicity of some variants detected by NGS. Full disclosure of the level of confidence in the clinical meaning of confirmed results, careful patient selection and informed consenting, with a clear understanding of the context in which NGS testing is sought, and genetic counseling before and after testing are important quality measures for the clinical use of NGS testing.

## 5. Physician Education and Training

It seems certain that the incorporation of genomic methods into clinical patient care will significantly change some aspects of the practice of medicine, affecting not only those who are performing and interpreting genomic testing, but virtually all healthcare professionals. Efforts to integrate genomic approaches, or approaches to analyze transcriptomes, proteomes, metabolomes, microbiomes,* etc*., into clinical care need to be paralleled by the education of our medical students, residents, clinical fellows and faculty to provide a fund of knowledge and an understanding of the possibilities, strengths and limitations of these approaches when they are translated from research to the bedside. This can be accomplished through core efforts in medical school curriculum design, residency and fellowship training programs, as well as in the form of informal and formal continuing medical education (CME) for practicing physicians. Virtually every medical specialty will need to incorporate genomics aspects, as they pertain to that specialty, into their education. A concerted educational effort in medical schools will be critical to ensure the appropriate application of genomic testing, and resources are beginning to become available from professional and medical specialty organizations (for example, the Training Residents in Genomics Working Group, [36]). 

Recently, several pathology residency programs have introduced curriculum changes to include more genomic medicine teaching [27,28,37]. Admittedly, in the face of all the other knowledge that residents must acquire in the course of their training, these initial efforts will not produce pathologists who are equally expert in bioinformatics, histology and the wide scope of other laboratory testing methods, but they do represent a first step toward systematic training in the interpretation of large DNA sequence data sets. 

## 6. Ethical and Privacy Considerations

### Quality of Patient Information and Informed Consent

Informed consent is the keystone of the ethical treatment of patients in clinical care settings. Patients must have the opportunity to learn about the benefits and limitations that may be associated with genomic testing and consider whether they wish to proceed with such tests. Especially because genomic methods (and other types of “omics” testing) can, in principle, provide magnitudes more information than those traditionally derived, the informed consent process can be more challenging. The level of that challenge depends on the scope of testing and is very different for single gene tests compared to the large net that can be cast with methods that determine the entire exome or genome of the patient. In the latter scenario, incidental findings may disclose medical or personal issues that the patient was not aware of and that were not part of the reason for the current care episode, but that may impact overall health and medical care. The process of returning incidental findings, especially when they may have direct medical ramifications, is an area of active discussion in the medical genetics community [38,39].

The ACMG has issued a policy statement emphasizing the need for informed consent and the content of such a process prior to exome or genome sequencing for germline conditions [40]. Additional recommendations by the ACMG addressing incidental findings have been released and updated [39]. The initial release of these recommendations gave rise to controversy about the informed consent process, and these issues are being considered by multiple additional medical specialties. Until community consensus is reached, individual institutions need to consider how to evaluate and communicate incidental findings of known significance, as well as those genome changes that are of, as yet, unknown clinical significance. What is reported, therefore, needs to be established upfront and clearly communicated to and meaningfully discussed with the patient or his or her guardian, so that expectations are accurate. An informed consent process includes patient education and counseling prior to test ordering, with information addressing the results that will be included in the patient’s report, as well as a discussion of the limits of genomic testing and the interpretation of sequence variants. In addition, as with any other testing, patients need to be able to rely on the privacy and confidentiality of their data. 

## 7. Guiding Principles for Clinical Genomic and Other “Omic” Testing 

Any medical center or healthcare organization seeking to incorporate genomic or other “omic” testing into its system of patient care would be well served by: (1) ensuring that the testing is performed in a manner that is fully in accord with relevant legal and regulatory requirements and by personnel with the appropriate training and credentials to perform and interpret such testing; and (2) involving representatives of medical specialties, researchers in genetics, statisticians, computer scientists, bioethicists and hospital administrators in the planning, implementation and integration of this new kind of testing into clinical decision-making. Some key principles we recommend for consideration are listed in Box 1 and are described below:

**Box 1. Guiding principles for clinical genomic and other “omic” testing:**

(a) Clinical laboratory testing is an integral component of patient care and is held to different standards than research testing not used to guide clinical care.
(b) Clinical genomic testing requires extra effort to be dedicated to designing the informed consent and patient education processes.
(c) Education of physicians and other care-givers about genomic testing methods will be critical for appropriate use and maximal patient benefit.
(d) The use of less-extensively validated genomic testing approaches for clinical care ordinarily should progress in a graded manner from use in “innovative care” settings, followed by use in clinical research settings, before being added to “standard” clinical laboratory testing.
(e) Individual and institutional conflicts of interest in clinical genomic testing must be identified and managed.
(f) These guiding principles also apply to efforts to introduce other clinical “omics” testing into clinical care (such as transcriptomes, proteomes, metabolomes and microbiomes).
(g) Clinical genomic and other “omic” data and methodologies should, to the greatest extent possible, be shared openly with the wider medical and research communities, to accelerate the pace of medical discovery and to increase the quality and reproducibility of clinical genomic data analysis.

*(a) Clinical Laboratory Testing Is an Integral Component of Patient Care and Is Held to Different Standards than Research Testing Not Used to Guide Clinical Care *
  Clinical laboratory testing, regardless of the assay methodology or test complexity, is done to guide patient care decisions, including making diagnoses, counseling patients regarding their prognosis or their future risk of developing disease, guiding management of the patient’s condition and making recommendations about reproductive or life style choices. Multidisciplinary committees of clinician specialists and clinical laboratory geneticists, guided by recommendations from medical specialty organizations, as well as other sources of information, may be best able to decide which new genomic tests or applications are sufficiently well-supported by evidence in the scientific literature to be adapted for clinical use.
  Any implementation of clinical genomic testing must, of course, comply fully with all relevant laws and regulations governing laboratory tests and, in the United States of America, meet the standards of the professional bodies, such as the College of American Pathologists (CAP) and/or ASHI (the American Society for Histocompatibility and Immunogenetics) that, together with The Joint Commission, have been deemed the status to inspect clinical laboratories on behalf of the Centers for Medicare and Medicaid Services (CMS) to ensure that requirements for CLIA certification are met and all medical tests, including genomic tests, are being carried out responsibly.
*(b) Clinical Genomic Testing Requires Extra Effort to be Dedicated to Designing the Informed Consent and Patient Education Processes *
  Patients must be able to obtain sufficient information about the potential value, future implications and limitations of genomic testing so as to be able to give informed consent if they choose to “opt-in” to the use of such tests for their care. Patient education about genomic test results will help to ensure that any subsequent clinical decision-making is carried out as an informed collaborative process between the patient and their physician. In many cases, this may require additional time to be spent by hospital personnel with the patient to ensure that they understand what is measured and what is interpreted from genomic tests. It is likely that the development of additional educational resources for patients will be necessary for this process. As with almost any other clinical interaction with patients, the use of genomic testing should be done only on the basis of a patient decision to “opt-in”, rather than as a default pathway from which patients need to “opt-out”.
*(c) Education of Physicians and Other Care-Givers about Genomic Testing Methods will be Critical for Appropriate Use and Maximal Patient Benefit *
  Medical centers and healthcare organizations should consider establishing a set of resources, including a service staffed by individuals with training in molecular genetic pathology, medical genetics and genetic counseling, to educate and advise medical personnel about the proper selection of genetic tests and the appropriate interpretation of their results. The need for such a resource has been highlighted by a recent study performed at a large reference laboratory, which documented a strikingly high rate of inappropriate selection of genetic tests (e.g., ordering the incorrect test, ordering tests that were not needed or ordering suboptimal tests given the clinical question being asked). Approximately 26% of all requests for complex genetic testing for heritable disease were changed following review [41]. These misorders result in unnecessary costs to the healthcare system and, more importantly, may result in significant clinical consequences (e.g., failure or delays in receiving necessary testing, receiving incidental or secondary findings that were not requested or desired).
  If clinical findings indicate that genomic tests could be helpful for the care of a particular patient, integrated “tumor board”-style meetings are particularly important in evaluating whether genomic sequencing methods should be applied for the care of that individual patient and for discussing the results and implications of the new genomic data for that patient’s care, particularly in challenging cases. It is still an open question as to how the cost of physician and other professional time and effort will be compensated for such diagnostic conferences, but the trend toward health system payments based on patient outcomes rather than the sheer volume of clinical work performed in a patient’s care may be compatible with such new efforts, if genomic testing contributes significantly to optimal diagnostic and management decisions and the cost-effectiveness of caring for individuals and populations in coming years.
*(d) The Use of Less-Extensively Validated Genomic Testing Approaches for Clinical Care Ordinarily Should Progress in a Graded Manner from Use in “Innovative Care” Settings, Followed by Use in Clinical Research Settings, before being Added to “Standard” Clinical Laboratory Testing *
  It is likely that a range of different approaches or applications of “genomic” testing will continue to be proposed by physician scientists and other medical investigators, spanning a wide range of different kinds of measurements and interpretations, with widely-varying levels of evidence for their actual clinical utility in different clinical contexts. There are preexisting good models for incorporating innovative clinical methods into practice, and these can be applied to the evaluation of genomic tests supported by various levels of prior evidence. For applications of “genomic” technology that measure already well-established genetic variants with clear clinical significance, typically by replacing older Sanger DNA sequencing assays, rapid incorporation into clinically and analytically validated molecular genetic pathology testing in the CLIA-certified clinical laboratories is advisable. The results of such sequence interpretation are applied for clinical decision-making in the same manner as equivalent results obtained using prior testing methods.
  When the clinical value of genomic testing is not well established, but few or no other adequate diagnostic testing options exist, then, as with other types of “innovative clinical care” adopted by medical centers, the application of these tools on very limited numbers of patients for specific purposes at the discretion of the clinician can be considered. If genomic testing will be applied systematically on multiple subjects, without established evidence of clinical utility, it should be carried out in the context of a clinical research study with the accompanying human subject protections and regulations associated with this activity. This would, of course, include obtaining informed consent from the patient for the research, following an explicit and detailed discussion of the limitations of the novel test as a basis for making clinical decisions or healthcare recommendations, the kinds of unexpected (and in some cases, unwanted by the patient) test results that could be reported to the patient and their physician and additional confirmatory testing that would be required before making clinical decisions based on the results of the novel test.
  As genomic testing and interpretation methods are validated by ongoing clinical research studies and evidence accumulates for the clinical utility of particular approaches in a given clinical context, some testing and interpretation methods would become sufficiently mature to join the list of “standard” laboratory tests that can be ordered for individual patients by clinicians without the additional safeguards and consultations described above. To the extent that patient informed consent permits, the data and interpreted results of genomic tests should be stored in databases that will permit additional research and discovery to proceed and derive additional clinical insights and knowledge from the testing process.
*(e) Individual and Institutional Conflicts of Interest in Clinical Genomic Testing must be Identified and Managed *
  Conflicts of interest are a serious concern for physicians and others responsible for patient care, and the current period of great discovery and commercial interest in clinical genomics has presented opportunities for physician-scientists and others to become involved in the commercialization of new genomic testing or interpretation methods. All physicians and healthcare institutions must be vigilant to ensure that such potential conflicts of interest do not lead to inappropriate decisions about the kinds of testing approaches to pursue or not to pursue. Individual conflicts of interest, where a faculty member or physician has a financial stake in a private company and/or intellectual property related to genomic testing and analysis methods, are similar to those that apply to the use of other medical technologies, pharmaceuticals and devices. Institutional conflicts of interest are those where the healthcare organization or those directing it could influence decisions about which kinds of diagnostic testing would be used for the care of patients, either to encourage the use of particular test methods, instruments, analytical systems or outsourcing to particular genomic testing to particular companies, or else the avoidance of particular tests or companies. Information about any such potential conflicts should be publicly available, as well as scrutinized and managed within the organization, to ensure and document the propriety and ethical behavior of all participants.
*(f) These Guiding Principles also Apply to Efforts to Introduce Other Clinical “Omics” Testing into Clinical Care (Such as Transcriptomes, Proteomes, Metabolomes and Microbiomes). *
  The improvement in NGS-based sequencing methods over the past five years has resulted in dramatic decreases in the cost per base of DNA sequence. Initially, these technologies revolutionized research endeavors, but clinical laboratories rapidly adopted these technologies. Other related complex testing using NGS or other technologies in the research setting shows significant potential clinical utility. These methods and technologies include RNA sequencing (complementary DNA sequencing), proteomics, metabolomics and metagenomics, as well as functional genomic studies. We suggest that these guiding principles for genomic testing can also be applied to the incorporation of other complex clinical laboratory testing into patient care when sufficient evidence is available to support clinical utility.
*(g) Clinical Genomic and Other “Omic” Data and Methodologies Should, to the Greatest Extent Possible, be Shared Openly with the Wider Medical and Research Communities, to Accelerate the Pace of Medical Discovery and to Increase the Quality and Reproducibility of Clinical Genomic Data Analysis *
  With many technological advances, there are opportunities for private enrichment, as well as the creation of new public resources. The balance between these two components can shape the pace of adoption and the ultimate impact of the technology; the history of the development of the Internet and the role of “open source” contributions to it show the key impact that communities with some element of altruism or public spiritedness can have on the success of a technology. The recent U.S. Supreme Court ruling in *AMP v*.* Myriad Genetics*,* Inc*. [42], which determined that human genes themselves are not able to be patented, and the preceding ruling in *Mayo Collaborative Services v. Prometheus Laboratories, Inc.* [43], which found that correlations between measured analytes and medical interpretations of such data do not qualify as patentable inventions, may have somewhat decreased the likelihood that private companies will attempt to use litigation to deter testing for particular human gene mutations [44]. These rulings may increase the likelihood that individual companies may try to amass, and keep in private hands, human genetic information and clinical interpretations as trade secrets. In spite of this possibility, there is now an opportunity for medical centers and other healthcare institutions to cooperate in sharing data, interpretations and analysis methods, to speed the identification of correlations between genomic sequences and disease risks, prognosis and treatment outcomes. Currently, the initial frameworks for such data sharing and coordination efforts are promising, but medical organizations and, particularly, their patients will benefit from further joint activity in the public domain that advances clinical genomics [45] and that can serve as a counter-balance to siloed, competitive, inward-looking efforts (whether in academic or commercial settings).

## 8. Conclusions

We have entered a new era of clinical testing, in which genetic data and other types of “omics” data are much more easily obtained, but the challenges of their interpretation will likely continue for many years. A balance between efficient adoption of new genomic tests and careful consideration of the reliability and clinical value of the results derived from genomic sequence data is needed as these methods become more widely disseminated and utilized within healthcare systems. There are key differences between the quality standards for assays used in research experimentation compared to those that must be maintained for clinical testing, and these standards are under active development and refinement by laboratory professional organizations, as well as associations focusing on particular clinical conditions or specialties. We have outlined seven key principles for consideration in implementing clinical genomic testing, encompassing the technical, as well as the human elements that must be engaged and coordinated to enable optimal utilization of this new form of clinical care. Above all, we feel that this period of intense exploration and discovery in human genetics represents a major opportunity for cooperative and transparent work between different areas of laboratory and clinical medicine, for the ultimate benefit of the patients.
